# Developing an affordable hyperspectral imaging system for rapid identification of *Escherichia coli* O157:H7 and *Listeria monocytogenes* in dairy products

**DOI:** 10.1002/fsn3.2749

**Published:** 2022-01-18

**Authors:** Phoebe Unger, Amninder Singh Sekhon, Xiongzhi Chen, Minto Michael

**Affiliations:** ^1^ School of Food Science Washington State University Pullman Washington USA; ^2^ Department of Mathematics and Statistics Washington State University Pullman Washington USA

**Keywords:** cheese, dairy, hyperspectral imaging, milk, pathogens, rapid identification

## Abstract

The objective of this foundational study was to develop and evaluate the efficacy of an affordable hyperspectral imaging (HSI) system to identify single and mixed strains of foodborne pathogens in dairy products. This study was designed as a completely randomized design with three replications. Three strains each of *Escherichia coli* O157:H7 and *Listeria monocytogenes* were evaluated either as single or mixed strains with the HSI system in growth media and selected dairy products (whole milk, and cottage and cheddar cheeses). Test samples from freshly prepared single or mixed strains of pathogens in growth media or inoculated dairy products were streaked onto selective media (PALCAM and/or Sorbitol MacConkey agar) for isolation. An isolated colony was selected and mixed with 1 ml of HPLC grade water, vortexed for 1 min, and spread over a microscope slide. Images were captured at 2000× magnification on the built HSI system at wavelengths ranging from 400 nm to 1100 nm with 5‐nm band intervals. For each image, three cells were selected as regions of interest (ROIs) to obtain hyperspectral signatures of respective bacteria. Reference pathogen libraries were created using growth media, and then test pathogenic cells were classified by their hyperspectral signatures as either *L. monocytogenes* or *E. coli* O157:H7 using *k*‐nearest neighbor (*k*NN) and cross‐validation technique in R‐software. With the implementation of *k*NN (*k* = 3), overall classification accuracies of 58.97% and 61.53% were obtained for *E. coli* O157:H7 and *L. monocytogenes*, respectively.

## Graphical abstract

The objective of this foundational study was to develop and evaluate the efficacy of an affordable hyperspectral imaging (HSI) system to identify single and mixed strains of foodborne pathogens in dairy products. With the implementation of kNN (*k* = 3), overall classification accuracies of 58.97% and 61.53% were obtained for *E. coli* O157:H7 and *L. monocytogenes*, respectively.

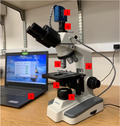

## 1 INTRODUCTION

Despite being one of the safest food systems, numerous food products in the United States are linked with foodborne illness, hospitalizations, and deaths (CDC, 2021). In developed countries like the United States, milk and milk‐based product outbreaks represent about 1%–6% of the total bacterial foodborne outbreaks (Claeys et al., 2013; Grace et al., 2020). Due to their unique composition and properties, milk and dairy products represent excellent growth media for many pathogens including *Escherichia coli* O157:H7 and *Listeria monocytogenes* (Cancino‐Padilla et al., 2017). The frequent involvement of these pathogens in product recalls and outbreaks has reinforced the need for their rapid detection and identification methods in foods. Although conventional methods are still used for detection and identification of foodborne pathogens due to being sensitive, inexpensive, and their ability to provide both qualitative and quantitative results (Doyle & Buchanan, 2013), these methods are time‐consuming, laborious, and can take from 4 to 7 days to give confirmatory results (FDA, 2021). Any method that can reduce the analysis time by a day, or even several hours compared to the conventional counterpart is considered a rapid test.

Hyperspectral imaging (HSI) is a novel technology in the field of food safety that has great potential in rapid, reliable, and inexpensive identification of foodborne pathogens. The HSI integrates conventional imaging and spectroscopy techniques to simultaneously gather both spatial (*x*‐ and *y* dimensions of image) and spectral (wavelength, λ) information of a sample to form a hyperspectral cube (Gowen et al., 2007). The hyperspectral cube can store vast amount of information in this three‐dimensional (3‐D) hyperspectral cube. In HSI, images of a specimen are acquired at various contiguous predefined wavelengths in the visible/near‐infrared region (approximately 400–1000 nm) at specific wavelength intervals. This results in dozens or hundreds of images, giving every pixel in a hyperspectral image its own spectrum or hyperspectral signature, over a contiguous wavelength range (Ariana & Lu, 2008). Hyperspectral cubes can be broken down to a single pixel, or a selection of a group of pixels known as a region of interest (ROI). The hyperspectral signature can then be used as a unique fingerprint for rapid identification of respective specimens. The HSI utilizes optical characteristics of the sample captured over a wide wavelength range for identification; therefore, HSI uses the interactions between light and the molecular structure of a sample for its identification.

The HSI was initially developed for remote sensing and has since been proven useful in a multitude of disciplines such as astronomy, agriculture, pharmaceuticals, and medicine (Gowen et al., 2007). In the food industry, HSI had been studied predominantly for food quality assessment, such as rapid detection of defects in agricultural products. Food ranging from fruits, vegetables, meat, fish, and grains have had HSI applied to assess water and fat content, spoilage, and damage, and for product quality grading (Chen et al., 2020; Codgill, 2004; ElMasry et al., 2012; ElMarsy et al., 2008; Heia et al., 2007; Naganathan et al., 2008; Qin, 2005).

In terms of food safety, most of the previous HSI research for bacterial detection has been conducted at macroscale using bacterial colonies, but minimal research has been conducted at the single bacterial cell level (Eady & Park, 2016a). Using HSI, Michael et al. (2019) were able to identify foodborne pathogens at a cellular level (various strains of *Cronobacter sakazakii*, *Salmonella* spp., Shiga toxin producing *Escherichia coli* (STEC), and *Listeria monocytogenes*) with accuracy ranging from 66.66% to 100% within the respective genera. Michael et al. (2019) also demonstrated that HSI could differentiate cells treated with lauric arginate (antimicrobial) from healthy, untreated cells within each bacterium with high accuracy. Eady et al. (2019) used HSI to classify *Salmonella* in chicken rinsate and obtained an accuracy of 81.8% and 98.5%, without and with image preprocessing techniques, respectively.

Currently, what is stopping researchers from exploring this technology is the initial startup cost of acquiring HSI systems. Most preassembled and preprogrammed HSI systems cost over $100,000 and have very little room for modification. The development of a cheaper and reliable HSI system by mounting a commercially available hyperspectral imaging camera on a regular laboratory compound microscope and using commercially available software could encourage other researchers to explore this technology and could invoke more interest from the food industry in investing in this technology.

The first objective of this study was to develop an affordable HSI system using a basic compound microscope and an HSI camera connected to a computer interface. The second objective was to evaluate the efficacy of the newly developed affordable HSI system to identify single and mixed strains of *L. monocytogenes* and *E. coli* O157:H7 in growth media, and selected dairy products (whole milk, and cottage and cheddar cheeses).

## 2 MATERIALS AND METHODS

### 2.1 Development of an affordable HSI system

**FIGURE 1:**
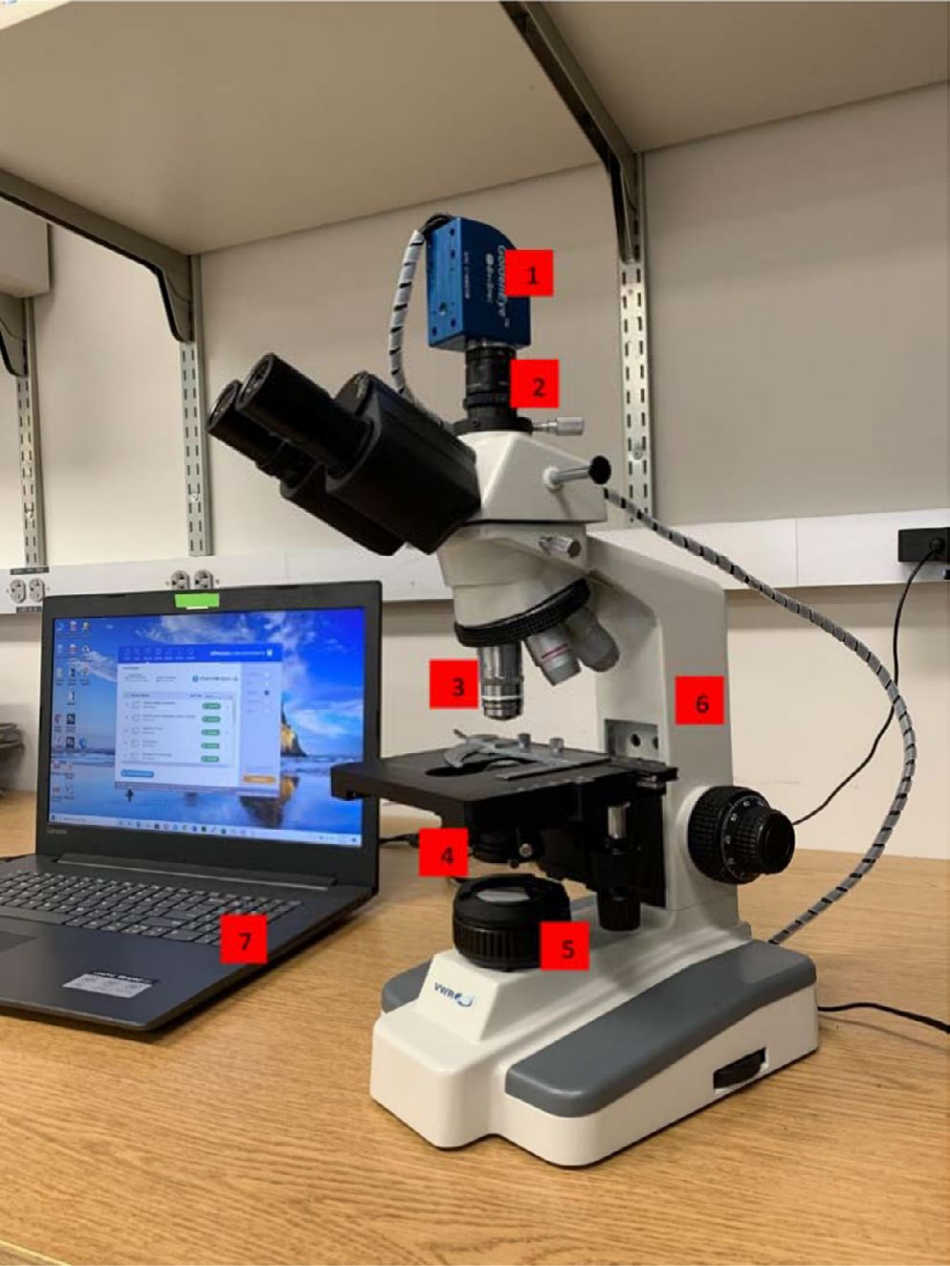
Affordable custom‐designed hyperspectral microscope imaging setup. 1: GoldenEye^TM^ hyperspectral imaging camera; 2: custom‐designed 20 × adapter; 3: adjustable numerical aperture 100 × objective; 4: dark field adapter; 5: halogen light source; 6: VWR compound microscope; and 7: computer

An affordable HSI system (Figure 1) was assembled by integrating a GoldenEye^TM^ snapshot hyperspectral imager (BaySpec Inc., San Jose, CA) with a B3‐223 trinocular microscope (VWR^®^ International, Radnor, PA). Several engineering adjustments were performed to transform the above‐mentioned technologies into one functional HSI system. Briefly, a dark field attachment (Motic^®^, Schertz, TX) was inserted below the condenser and above the halogen lamp of the microscope. A 100× oil dark field plan objective with an adjustable iris (AmScope^TM^, Irvine, CA) was installed on the revolving nose piece of the microscope. A custom‐designed 20× adapter (Engineering Shop, Washington State University, Pullman, WA) built by installing a wide field 20× lens into a 3‐D printed adapter was used to connect the hyperspectral imager directly to the trinocular port on the microscope. Lastly, the hyperspectral imager was connected through a USB (universal serial bus) port to a laptop computer (Dell^®^, Round Rock, TX). The price breakdown of the completed affordable custom‐designed HSI setup is shown in Table 1.

**TABLE 1:** Approximate price breakdown of the affordable custom‐designed hyperspectral imaging setup

Item	Company	Price (U.S. dollars)
Trinocular compound microscope	VWR^®^ International	$1500
Dark field attachment	Motic^®^	$48
100 × oil dark field microscope plan objective w/ iris	AmScope^TM^	$345
Custom‐designed 20 × adapter	Engineering Shop, WSU	$140
GoldenEye^TM^ Snapshot Hyperspectral Imager	BaySpec Inc.	$16,450
Laptop computer	Dell^®^	$480
ENVI license, tech support, and maintenance	Harris Geospatial Solutions Inc.	$1983
Total cost		$20,946

### 2.2 Experimental design

This study was designed as a completely randomized design. To study whether the custom‐designed affordable HSI system can be used for the rapid identification of various foodborne pathogens, three strains each of *Escherichia coli* O157:H7 and *Listeria monocytogenes* were used. Hyperspectral images of immobilized cells from isolated colonies were captured. These images were then used to generate hyperspectral graphs of respective bacterial cells. The hyperspectral graphs/signatures of cells of pure cultures obtained from respective selective agar were stored as a reference library and used to train the classification model. The *k*‐nearest neighbor (*k*NN) classifier (with an optimal *k* determined by cross‐validation) was used to classify unknown bacterial cells (*Escherichia coli* O157:H7 and *Listeria monocytogenes*) from artificially inoculated dairy products. Three replications were conducted for both pure cultures and inoculated dairy product samples for generating hyperspectral graphs; and within each replication, hyperspectral images of various samples were obtained randomly.

### 2.3 Culture propagation

*Escherichia coli* O157:H7 and *L*. *monocytogenes* strains used in this study are presented in Table 2. All strains were selected on the basis of risk and involvement in foodborne disease outbreaks or isolated from the environment and food processing facilities. The cultures were propagated according to manufacturers’ instructions, individually transferred onto glycerol protectant beads (Microbank^TM^, Richmond Hill, ON), and stored in a −80°C freezer (Panasonic Healthcare Co., Ltd., Wood Dale, IL) until used. At the start of the study, a frozen bead of each culture was grown individually at 37°C for 24 h in 10 ml of brain heart infusion (BHI) broth (Difco^TM^, Becton, Dickinson and Company, Sparks, MD) and stored at 4˚C as stock cultures. All stock cultures were confirmed using API^®^ 20E and API^®^ *Lister* (bioM´erieux, Inc., Durham, NC) for *E. coli* O157:H7 and *L. monocytogenes* strains, respectively.

**TABLE 2:** Bacterial cultures used in the study

Pathogen	Strain no.	Isolated from	Source
*Escherichia coli* O157:H7	905	Human	(Sheng et al., 2006) University of Idaho (Moscow, ID)
	35,150	Human	ATCC
	43,895	Raw hamburger meat outbreak	ATCC
*Listeria monocytogenes*	5414	Raw milk outbreak in Massachusetts	ATCC
	19,111	Poultry, England	ATCC
	19,115	Human	ATCC

Abbreviation: ATCC^®^: American Type Culture Collection (Manassas, VA).

### 2.4 Sample preparation using selective media

For each replication, a loop of an individual stock culture was used to inoculate 10 ml of BHI broth and incubated at 37°C for 24 h. Incubated strains (8.3 ± 0.07 and 8.0 ± 0.05 log CFU/ml, for *E. coli* O157:H7 and *L. monocytogenes* strains, respectively) were individually streaked for isolation on MacConkey (Criterion, Hardy Diagnostics, Santa Maria, CA) or PALCAM (Difco, Becton, Dickinson and Company, Sparks, MD) agar for *E. coli* O157:H7 and *L. monocytogenes*, respectively, and incubated at 37°C for 24 h. A mixed‐culture cocktail (containing one strain of *E. coli* O157:H7 and one strain of *L. monocytogenes*) was prepared by mixing 1 ml of freshly, individually grown *E. coli* O157:H7 (strain 35,150) and 1 ml of freshly, individually grown *L. monocytogenes* (strain 19,111), vortexed for 1 min, and was streaked on MacConkey and PALCAM agar and incubated at 37°C for 24 h.

### 2.5 Dairy product preparation and inoculation

Dairy products (milk and cheeses) were purchased from a local store in Pullman, WA (Walmart, Pullman, WA). For each replication, a loop of individual stock cultures was used to inoculate 10 ml of BHI broth and incubated for 37°C for 24 h. As previously described in Section 2.4, a mixed‐culture cocktail was also prepared by mixing 1 ml of freshly, individually grown *E. coli* O157:H7 (strain 35,150) and 1 ml of freshly, individually grown *L. monocytogenes* (strain 19,111), vortexed for 1 min. Dairy products were inoculated with individual cultures (8.3 ± 0.07 and 8.0 ± 0.05 log CFU/ml, for *E. coli* O157:H7 and *L. monocytogenes* strains, respectively), as well as the mixed‐culture cocktail. For each replication, 10 ml of whole milk was inoculated with 0.1 ml of culture, 20 g of cottage cheese was inoculated with 0.2 ml of culture, and a slice (45.16 cm^2^) of cheddar cheese was inoculated with a swab of respective cultures. All inoculated dairy samples were refrigerated at 4˚C for 24 h. After incubation, the cheddar cheese samples were stomached for 1 min with 20 ml of 0.1% peptone water (Bacto^TM^, Becton, Dickinson and Company, Sparks, MD), cottage cheese samples were stomached for 1 min, and milk samples were vortexed for 1 min. A sterile cotton swab (Puritan^®^, Guilford, ME) was used to streak for isolation on the respective selective agar for individual cultures (MacConkey or PALCAM) and was incubated at 37°C for 24 h. For mixed cultures, products were streaked on both MacConkey and PALCAM agar, and were incubated at 37°C for 24 h and 48 h, respectively.

### 2.6 Bacterial slides preparation

Bacterial cells from individual pure cultures, mixed cultures, or inoculated dairy products were obtained by just touching a loop (0.01 ml) (VWR^®^ International, Radnor, PA) on an isolated colony on the respective agar plates and mixing in 1 ml of filtered (0.2 µm) sterilized HPLC grade water (J.T. Baker Inc., Phillipsburg, NJ) in microcentrifuge tubes by vortexing for 1 min. For each HSI analysis, a loop of vortexed cell solution was transferred onto a clean sanitized 1‐mm glass slide (Fisherbrand, Fisher Scientific, Pittsburgh, PA) and immobilized by air drying in a biosafety cabinet for 5 min. These immobilized bacterial cells on glass slides were then used for HSI analysis.

### 2.7 Hyperspectral graph generation

The custom‐designed HSI microscope system was used to capture hyperspectral images. Environment for Visualizing Images (ENVI) (Harris Geospatial Solutions Inc., Boulder, CO) software version 5.6 was used for analyzing acquired hyperspectral images and generating hyperspectral graphs. Hyperspectral images of individual bacterial cells on air‐dried glass slides were acquired by focusing the microscope at 2000× magnification and ENVI settings at 50 ms exposure time, and 0 gain. Hyperspectral images were acquired using the “snapshot” technique, which captures all spatial and spectral data simultaneously within one exposure, without any sample or detector movement. Using ENVI, three bacterial cells were selected from the acquired image as regions of interest (ROIs). Average scattering values at respective wavelengths of these three ROIs were used to generate hyperspectral graphs at wavelengths ranging from 400 to 1100 nm (at wavelength intervals of 5 nm resulting in 141 wavelength bands).

### 2.8 *k*NN classification and validation of hyperspectral graphs

An important step in statistical analyses and classification of a spectral data set is preprocessing; however, there are currently no well‐established guidelines or rules for selecting a particular preprocessing technique for a specific type of data set (Scott et al., 2006). The preprocessing technique chosen for a particular data set should aim to provide the best possible classification accuracy. For this study, hyperspectral graphs were preprocessed by normalizing the y‐axis (scattering value) from 0 to 1 (Michael et al., 2019; Scott et al., 2006), with “1” being the brightest point on the ROI and “0” being the darkest point. The following equation was used to calculate normalized scattering values (Michael et al., 2019; Scott et al., 2006):

$$y_{\mathrm{ij}}=\left[x_{\mathrm{ij}}‐\min\left(X_{j}\right)\right]/\left[\max\left(X_{j}\right)‐\min\left(X_{j}\right)\right]$$

where **X_j_** is a numeric vector and is the hyperspectral signature of the *j*th observation, *x_ij_* the *i*th entry of **X_j_** and is the scattering value at the *i*th wavelength; min (X_j_) is the minimum scattering value of the hyperspectral signature **X_j_**; and max (X_j_) is the max scattering value of the hyperspectral signature **X_j_**.

The *k*‐nearest neighbor (*k*NN) classifier was used to classify the different pathogens using the normalized hyperspectral signatures, where the value of neighboring size *k* was chosen to be 3 (as explained below). The *k*NN classifier is a commonly used, nonparametric classification technique. This classification technique was chosen since each (normalized) hyperspectral signature in this study is a high‐dimensional vector but there were only a few such signatures whose pathogen types were available to train a parametric classifier different than the *k*NN classifier. Specifically, the Euclidean distance between a pair of normalized hyperspectral signatures was used as a dissimilarity measure on these signatures. In order to determine an optimal neighboring size *k,* a training set was created and consisted of 18 normalized hyperspectral signatures of known pathogens, and a 5‐fold cross‐validation was applied to the training set. This gave an optimal value for *k* as 3. It should be noted that values for *k* from 1 to 3, and 5‐ or 10‐fold cross‐validation are commonly used, and considerable empirical evidence shows that these two choices of the number of folds for cross‐validation work well in practice for a range of statistical learning methods including *k*NN classifiers (James et al., 2013; Scott et al., 2006). For this study, 5‐fold cross‐validation was chosen in order to more stably estimate the test error of an optimal *k*NN classifier since the training set had only 18 observations. The optimal 3‐NN classifier was then applied to classify a total of 78 normalized hyperspectral signatures, all different from those in the training set, to classify them into their corresponding pathogens.

## 3 RESULTS

**FIGURE 2:**
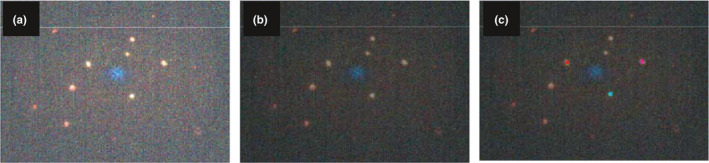
Hyperspectral images of *Escherichia coli* O157:H7 cells at 2000 × magnification. (a) Hyperspectral image as it appears under the field of view of the microscope in Environment for Visualizing Images (ENVI). (b) Hyperspectral image after preprocessing technique has been applied in ENVI. (c) Hyperspectral image with three regions of interest (ROIs) selected (pink, red, and blue) in ENVI

An example of the hyperspectral image of bacterial cells as visible under the field of view of the microscope and acquired by the affordable custom‐designed HSI system is presented in Figure 2a. Using the ENVI software, this image was clarified using various image‐clarifying tools for better visualization of bacterial cells presented in Figure 2b; however, clarification of the images did not affect the hyperspectral signatures of these bacterial cells. The selection of the three individual cells as ROIs within the hyperspectral image is presented in Figure 2c.

**FIGURE 3:**
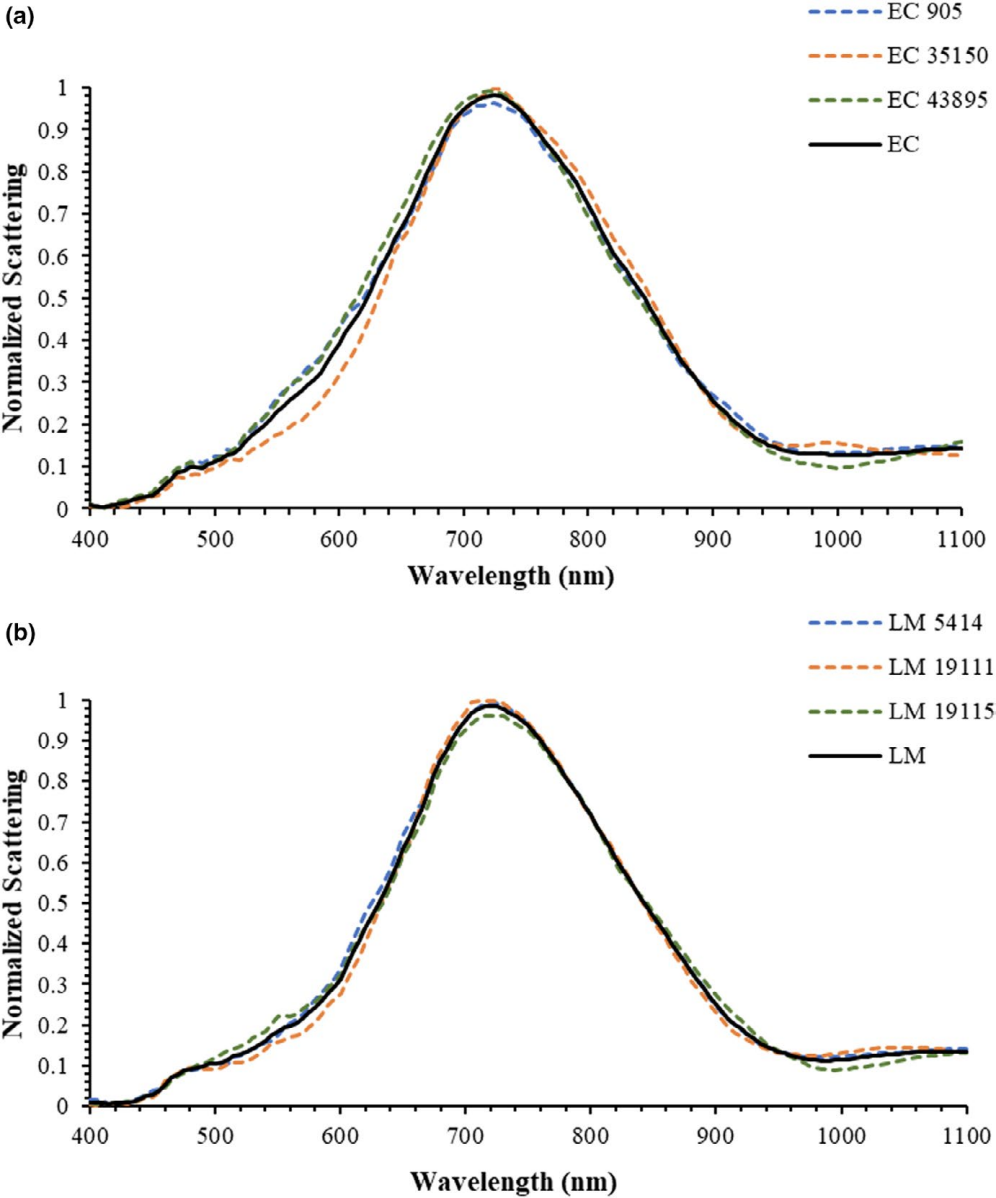
Hyperspectral graphs of: (a) individual *Escherichia coli* (EC) O157:H7 strains 905, 35,150, and 43,895 (dotted curves), and mean of all *E. coli* O157:H7 strains (continuous curves); (b) individual *Listeria monocytogenes* (LM) strains 5414, 19,111, and 19,115 (dotted curves), and mean of all *L. monocytogenes* strains (continuous curves) captured at wavelength ranging from 141 wavelengths from 400 to 1100 nm (with 5‐nm band intervals)

To evaluate the efficacy of the assembled HSI system, hyperspectral images of different reference strains of *E. coli* O157:H7 and *L. monocytogenes* grown in nutrient growth media followed by isolating on selective agar were captured to develop a training data set to train the *k*NN classification model. The mean hyperspectral graphs from 400‐ to 1,100‐nm wavelength range for these reference strains are presented in Figure 3. The graphs presented in Figure 3 demonstrated that there were overall differences in scattering intensities in the hyperspectral graphs of *E. coli* O157:H7 and *L. monocytogenes*. The main difference between *E. coli* O157:H7 and *L. monocytogenes* was observed in the wavelength range of 500–700 nm, with the scattering intensities of *L. monocytogenes* being lower than those of *E. coli* O157:H7. Likewise, *L. monocytogenes* has lower scattering intensities at the wavelength range of 900–1025 nm in comparison to those of *E. coli* O157:H7.

The graphs in Figure 3 also demonstrate that the different strains within each genus vary slightly. The scattering intensity for *E. coli* O157:H7 strain 35,150 was lower at wavelength range 400–705 nm and higher at wavelength ranges 710–885 nm and 945–1035 nm, compared to the other two *E. coli* O157:H7 strains. However, the scattering intensity for *E. coli* O157H7 strain 43,895 was higher at wavelength range 415–750 nm and was lower at wavelength range 885–1070 nm, compared to the scattering intensities of the *E. coli* O157:H7 strains 35,150 and 905. Among the *L. monocytogenes* strains, the scattering intensity of *L. monocytogenes* strain 19,111 was lower in the wavelength range 490–655 nm and was higher in the wavelength ranges 665–835 nm and 960–1085 nm, compared to the scattering intensities of the other two *L. monocytogenes* strains.

The confusion matrix of the optimal 3‐NN classifier of the inoculated dairy products and mixed cultures on respective selective agar is presented in Table 3. A confusion matrix is a table that is used to describe the performance of a classifier, such as *k*NN, on a set of test data for which true values are known. For mixed cultures grown on the respective selective agar and all the dairy products that were either single or mixed inoculated (as described in Sections 2.4 and 2.5), the classification accuracies of *E. coli* O157:H7 and *L. monocytogenes* were 58.97% and 61.53%, respectively. In other words, out of the 78 total (39 of each pathogen) hyperspectral images, 23 *E. coli* O157:H7 and 24 *L. monocytogenes* images were classified accurately as the respective pathogens (Table 3). The classification results are impressive, since this study had only 18 reference/standard hyperspectral signatures of pure cultures in the training set to build the optimal classifier but applied it to classify a total of 78 normalized hyperspectral signatures.

**TABLE 3:** Confusion matrix for the optimal *k*NN classifier (with k = 3) to classify 78 bacterial hyperspectral images as *Escherichia coli* O157:H7 (EC) and *Listeria monocytogenes* (LM) using their normalized hyperspectral signatures that were obtained from the custom‐designed hyperspectral imaging system from 400 to1100 nm (with 5‐nm band intervals)

*Predicted*	*Actual*	
	EC	LM
EC	23^a^	15
LM	16	24^a^

^a^ 23 out of 39 EC samples and 24 out of 39 LM samples were classified correctly.

A breakdown of the classification results from the optimal *3‐*NN classifier of the inoculated dairy products is presented in Table 4. Overall, cheddar cheese had the highest classification accuracy for *E. coli* O157:H7 at 66.67% compared to whole milk and cottage cheese with accuracies of 58.33% and 50%, respectively. However, overall, whole milk and cottage cheese both had higher classification accuracies for *L. monocytogenes* at 66.67% compared to cheddar cheese at 50%. The *E. coli* O157:H7 strain 43,895 had the worst classification accuracy in all three dairy products, with an accuracy of 33.33% in whole milk and cheddar cheese, and 0% accuracy in cottage cheese.

**TABLE 4:** Classification accuracy of various strains of *Escherichia coli* O157:H7 and *Listeria monocytogenes* in dairy products (whole milk, cottage and cheddar cheeses) obtained from the optimal *k*NN (*k*‐nearest neighbor) classifier with optimal *k* = 3

Product	Bacteria	Strain	% Classification accuracy	Total % classification accuracy
Whole milk	*Escherichia coli* O157:H7	905	66.67	
		35,150	66.67	
		43,895	33.33	
		Mixed (35,150)	66.67	58.33
	*Listeria monocytogenes*	5414	66.67	
		19,111	33.33	
		19,115	100	
		Mixed (19,111)	66.67	66.67
Cottage cheese	*Escherichia coli* O157:H7	905	66.67	
		35,150	66.67	
		43,895	0	
		Mixed (35,150)	66.67	50
	*Listeria monocytogenes*	5414	66.67	
		19,111	100	
		19,115	33.33	
		Mixed (19,111)	66.67	66.67
Cheddar cheese	*Escherichia coli* O157:H7	905	66.67	
		35,150	66.67	
		43,895	33.33	
		Mixed (35,150)	100	66.67
	*Listeria monocytogenes*	5414	33.33	
		19,111	66.67	
		19,115	66.67	
		Mixed (19,111)	33.33	50

## 4 DISCUSSION

In a similar study, Michael et al. (2019) used HSI over the wavelength of 425.57–753.84 nm (with 1.29‐nm wavelength separation) for the identification and differentiation of four strains of *Cronobacter sakazakii*, five strains of *Salmonella*, eight strains of *Escherichia coli*, and a single strain *of Listeria monocytogenes* and *Staphylococcus aureus* at a cellular level using a commercial CytoViva HSI microscope setup. The author used principal component analysis and *k*NN classifiers to classify the hyperspectral signatures. Michael et al. (2019) reported 100% classification accuracy in all but four strains when analyzed within their respective genera. However, the overall classification when all strains of different genera were analyzed together was not as accurate, leading to 100% classification in only five of the nineteen strains.

Eady et al. (2019) investigated and compared the ability of visible/near‐infrared HSI with real‐time PCR to classify *Salmonella* in chicken rinsate. Spectral signatures between 450 and 800 nm from 341 images of bacterial cells from chicken rinsate samples were acquired with a hyperspectral microscope imaging system. Quadratic discriminant analysis (QDA) was performed to classify cells as either *Salmonella* positive or *Salmonella* negative. The classification accuracy of the system was 81.8%, but with the application of preprocessing techniques the classification accuracy rose to 98.5%.

Park et al. (2019) used hyperspectral microscope imaging at 450–800 nm to classify six bacteria: *Bifidobacter longum*, *Campylobacter jejuni*, *Clostridium perfringens*, *Enterobacter cloacae*, *Lactobacillus salivarius*, and *Shigella flexneri*, at a cellular level using their scattering intensities from the hyperspectral spectra. The overall accuracy of the QDA technique for classifying all six bacterial species was found to be 89% (Park et al., 2019).

Eady and Park (2016b) used metal halide and quartz halogen light sources within a hyperspectral microscope setup to obtain hyperspectral signatures of *Salmonella* serovars; *Enteritidis* and *Typhimurium* at a cellular level. The hyperspectral microscopic images were captured at the wavelength ranges from 450 to 800 nm, at 4‐nm spectral intervals with exposure time of 250 ms and gain value of 9. The *Salmonella* serovars were grown on BGS (Brilliant Green Sulfa) and XLT4 (Xylose‐Lysine‐Tergitol4) agar plates and then colonies were suspended in filter‐sterile phosphate‐buffered saline (PBS) and utilized for slide preparation. The spectral peaks for respective *Salmonella* serovars were similar within the respective light sources, with *Enteritidis* and *Typhimurium* peaking at 458, 498, 546, 590, and 670 nm when metal halide was used as light source. Likewise, the spectral peaks for respective serovars were obtained at 558, 646, 702, and 772 nm when halogen light source was employed for capturing the images. In comparison, the hyperspectral graphs or spectral peaks of the same bacterial cell obtained using two different light sources were clearly different, demonstrating the variation induced by the type of light source being employed. Furthermore, the research studies by Michael et al. (2019) and Eady et al. (2019) in comparison with this current study substantiate the point of introducing a variation in hyperspectral signatures of respective pathogens, either due to the different hyperspectral imaging setup or due to light sources being employed. The decreased classification accuracies from previous studies could also be attributed to differences in wavelength sampling intervals (Martinez et al., 2019). In previous studies, Eady et al. (2019), Eady and Park (2016b), and Michael et al. (2019) performed classification using 4‐nm, 2‐nm, and 1.29‐nm wavelength sampling intervals, respectively; however, this study used 5‐nm wavelength intervals.

Several limitations of this system, and within the field of hyperspectral microscope imaging, include the requirement of prior hyperspectral information about the target organism and an enrichment step. First, a reference library containing the target organisms’ hyperspectral signature is required to identify the unknown target organism (Gomez, 2002). Without previous hyperspectral information, such as a reference library, the classification technique would not be able to identify the unknown organism. Second, an enrichment step is necessary when looking to identify possible target organisms in food matrices (Bari & Yeasmin, 2015). The necessity of the enrichment step is due to the low concentrations of pathogens in foods and variability in cellular morphology due to various stresses encountered in the foods, such as pH, temperatures, and antimicrobials.

Currently, preassembled and preprogrammed HSI systems are expensive and can easily cost over $100,000, leading to limited research being explored using hyperspectral microscope imaging for the identification and detection of foodborne pathogens. However, with the introduction of an affordable HSI microscope setup built in this study, the initial investment cost is reduced to a fifth of the average cost of commercially available HSI systems. After the initial cost of the HSI system, the cost of running HSI analysis is considerably low, which would include the cost of enrichment and isolation media along with regular microbiological tools (such as loops, glass slides coverslips, and a biosafety cabinet). Once the bacterial colonies are isolated on appropriate agar, the time for the hyperspectral image acquiring and analyzing is less than 15 min. After developing an affordable, accurate, and precise HSI system, the HSI of the bacterial cells could be used as a rapid identification technique for specific pathogens or bacteria, at least at the presumptive levels.

In conclusion, the overall classification accuracy (60.25%) of this affordable custom‐designed HSI system with *k*NN classification model still needs improvement to be considered a reliable detection and identification method for foodborne pathogens within a food matrix. Further work will be performed to improve the bacterial cells’ magnification and classification accuracy of the HSI system. Likewise, different preprocessing and classification methods will be examined to increase the classification accuracy. Future research will focus on building a stronger reference library for more pathogens such as *Salmonella*, Big Seven Shiga toxin producing *E. coli* (STEC), *L. monocytogenes*, and *Staphylococcus aureus*. Once a stronger reference library has been established, future research will focus on the identification of pathogens within different food matrices such as low moisture foods. This technology has shown promising results with *E. coli* O157:H7 and *L. monocytogenes*, and the development of diverse standard library in future and enhanced magnification will make this even more potent. Overall, HSI has a bright future for its application in food safety.

## CONFLICT OF INTEREST

The authors do not have any conflict of interest.

## ETHICAL APPROVAL

This study does not involve any human or animal testing.
